# RCorp: a resource for chemical disease semantic extraction in Chinese

**DOI:** 10.1186/s12911-019-0936-3

**Published:** 2019-12-05

**Authors:** Yueping Sun, Li Hou, Lu Qin, Yan Liu, Jiao Li, Qing Qian

**Affiliations:** Institute of Medical Information, Chinese Academy of Medical Sciences/Peking Union Medical College, Beijing, 100020 China

**Keywords:** Corpus annotation, Chemical-disease relations, Chronic diseases, Combination therapy, Relation extraction

## Abstract

**Background:**

To robustly identify synergistic combinations of drugs, high-throughput screenings are desirable. It will be of great help to automatically identify the relations in the published papers with machine learning based tools. To support the chemical disease semantic relation extraction especially for chronic diseases, a chronic disease specific corpus for combination therapy discovery in Chinese (RCorp) is manually annotated.

**Methods:**

In this study, we extracted abstracts from a Chinese medical literature server and followed the annotation framework of the BioCreative CDR corpus, with the guidelines modified to make the combination therapy related relations available. An annotation tool was incorporated to the standard annotation process.

**Results:**

The resulting RCorp consists of 339 Chinese biomedical articles with 2367 annotated chemicals, 2113 diseases, 237 symptoms, 164 chemical-induce-disease relations, 163 chemical-induce-symptom relations, and 805 chemical-treat-disease relations. Each annotation includes both the mention text spans and normalized concept identifiers. The corpus gets an inter-annotator agreement score of 0.883 for chemical entities, 0.791 for disease entities which are measured by F score. And the F score for chemical-treat-disease relations gets 0.788 after unifying the entity mentions.

**Conclusions:**

We extracted and manually annotated a chronic disease specific corpus for combination therapy discovery in Chinese. The result analysis of the corpus proves its quality for the combination therapy related knowledge discovery task. Our annotated corpus would be a useful resource for the modelling of entity recognition and relation extraction tools. In the future, an evaluation based on the corpus will be held.

## Background

Relations between chemicals (drugs) and diseases (Chemical-Disease Relations or CDRs) play critical roles in drug discovery, biocuration, pharmacovigilance, etc. [1]. Combination therapies of drugs [2], disease treatments with two or more drugs, have the potential to improve efficacy while limiting toxicity. Various studies have demonstrated that a drug combination therapy may be beneficial in the treatment and management of chronic medical conditions, such as diabetes mellitus, Alzheimer’s disease, rheumatoid arthritis and pulmonary disorders [3–5], which are now among the most common and costly health problems worldwide [6, 7]. To robustly identify synergistic combinations, high-throughput screenings are desirable [8]. And mounting clinical evidence in biomedical text can help knowledge discovery of combination therapy for chronic diseases. It will be of great help to identify the relations in the published papers. However, the relation discovery/extraction process should be assisted by text mining tools due to the significant increases of the amount of biomedical text. Although some Information Extraction (IE) research has focused on unsupervised methods of developing systems [9, 10], most practical modern IE work requires data that have been manually annotated with the events, entities and relationships that are considered to express key content for the given domain [11].

Much effort have been done to manually curate entities and their relations. Roberts et al. [11] constructed a semantically annotated corpus of 150 clinical texts from the textual component of patient records which includes condition, intervention, drug, locus and their interaction relations. I2B2 [12] organized a challenge on concepts, assertions, and relations in clinical text and released a corpus with 871 annotated clinical records. The annotation framework of I2B2 is similar to the work of Roberts but with more becoming designs. And the I2B2 corpus focused on medical problem concepts and a relation classification task focused on assigning relation types that hold between medical problems, tests, and treatments. The medical problem in the I2B2 corpus includes diseases and symptoms, which are separately treated in some other researches. The above mentioned work are mainly based on clinical text, and some other efforts have been made based on scientific literatures. EU-ADR [13] constructed a corpus annotated for drugs, disorders, genes and their inter-relationships. For each of the drug–disorder, drug–target, and target–disorder relations three experts have annotated a set of 100 abstracts. To investigate the semantic relationships in biomedical texts, Rosario et al. [14] extracted sentences from titles and abstracts of Medline 2001 articles, and distinguished seven relation types that can occur between the entities “treatment” and “disease” in bioscience texts. The limitation of the corpus is that only relations within a sentence provided while biosciences texts are more likely to be composed of a number of sentences or a paragraph. Comparative Toxicogenomics Database provides manually curated 254,173 toxicogenomic interactions (152,173 chemical-disease, 58,572 chemical-gene, 5345 gene-disease and 38,083 phenotype interactions [15]. But the entity annotations, which are key features for machine learning tasks, are lacked. BioCreative V developed a corpus for both named entity recognition and chemical-disease relations in the literature. A total of 1500 articles have been annotated with automated assistance from PubTator [16, 17]. However, the combination therapies are recorded as several separate chemicals. To promote the performance of clinical named entity recognition on the Chinese clinical text, the 2017 and 2018 China conference on knowledge graph and semantic computing (CCKS) organized a named entity recognition (NER) evaluation task to identify and extract the anatomy, symptom, independent symptom, drug and operation from Chinese clinical text [18]. But no semantic relations among the entities released in the corpus.

In a word, existing corpora with CDR cannot support the chemical disease semantic relation extraction especially for chronic diseases in Chinese, while the annotation frameworks are useful for reference, especially the one of BioCreative V CDR. Therefore, to support the chemical disease semantic relation extraction especially for chronic diseases, a Chinese biomedical semantic relation corpus (RCorp) is manually annotated with a guideline clarifying combination therapies. The corpus aims to provide a standard dataset for the modelling of natural language processing tools, which mine knowledge about combination therapy of chronic diseases from biomedical text. In future, the mined CDR relations could be further visualized to enhance reading efficiency of researchers.

## Methods

To construct a corpus for chemical disease semantic extraction in Chinese, we followed the annotation framework of the BioCreative CDR corpus [16], with the guidelines modified to make the combination therapy related relations available.

### Article selection

In our work, we selected a famous Chinese Medical Server (WANFANG MED ONLINE) as the source of biomedical abstracts. The topics of RCorp articles were predefined to be limited to a number of typical chronic diseases including asthma, chronic obstructive pulmonary disease, tuberculosis of intestines, hypertension, diabetes mellitus, thyroadenitis, hepatitis, Sjogren’s syndrome, cerebral stroke, systemic sclerosis, chronic kidney disease, indolent lymphoma and leucocythemia.

According to those topics, 1000 articles were downloaded from the WANFANG MED ONLINE (http://www.wanfangdata.com.cn/index.html). Further, the titles and abstracts, which have more than one entity type and at least one entity interaction were recorded. To make sure the coverage of combination therapies, a total of 339 articles were finally filed to the dataset. Detailed disease topic distributions of the dataset are shown in Table 1. The disease topic distributions are rather scattered while popular chronic diseases like diabetes mellitus get larger proportions.

**Table 1:** The topic distributions of RCorp

Disease Topic	Proportion (percentage)
diabetes mellitus	16.2
leukemia	12.7
asthma	10.9
hypertension	11.5
chronic cardiopulmonary disease	10.0
myocardial infarction	8.26
cerebral infarction	4.72
hepatitis	3.83
thyroiditis	2.36
eye disease	2.36
intestinal tuberculosis	1.47
others	22.2

### Annotation tasks

The knowledge about combination therapies can be indicated by the chemical, disease and symptom entities and their relations. In the selected articles, few symptoms are mentioned and almost all of the chemicals are used to treat diseases rather than symptoms. Therefore, the relation annotation are defined as chemical-induce-disease, chemical-induce-symptom and chemical-treat-disease. And the chemical-treat-disease is the key relation in our work. We performed manual annotation of all chemical, diseases, symptoms and their interactions mentioned in the articles. For each entity occurrence, we not only annotated its text span but also assigned a relevant concept identifier from the Chinese Medical Subject Headings (CMeSH) [19], a controlled vocabulary of biomedical concepts provided by the Institute of Medical Information, Chinese Academy of Medical Sciences.

### Annotators

We recruited three CMeSH indexers, all of whom had a medical training background and curation experience. Each article was annotated independently by two annotators (i.e., double annotation). Differences were resolved by a third and senior annotator.

### Annotation guidelines

The task organizers followed the usual practice of biomedical corpus annotation for entity annotation and entity relation annotation. An important difference in the entity and relation annotation guideline is that the combination therapy should be annotated as a single mention to provide more hints to the relation recognition. In BioCreative CDR, a combination of chemicals should be annotated as two separate mentions of chemicals, and thus two separate relation mentions are annotated. That is, the combination therapy information is missed in the final annotated results, which is important for combination therapy related knowledge discovery. Therefore, to make the combination therapy information explicit, RCorp provides an alternative expression of chemical combinations by annotating the “AND” relations of chemicals in a combination therapy both on the entity level and on the relation level. For example, “培美曲塞联合顺铂” (Pemetrexed combined with Cisplatin) should be annotated as an entry “C0210657 C1859690” in the sentence “培美曲塞联合顺铂治疗非小细胞肺癌29例临床评价” (Clinical Evaluation on Pemetrexed Combined with Cisplatin in Treating 29 Patients with Non-Small Cell Lung Cancer), and a relation of chemicals combination “Pemetrexed combined with Cisplatin” treats disease “Non-Small Cell Lung Cancer” should be accordingly annotated as a single relation mention “C0210657 C1859690 CTD C0007131” rather than a combination of “C0210657 CTD C0007131” and “C1859690 CTD C0007131”, where “CTD” is the abbreviation of “chemical-treat-disease”.

If there are two individual mentions of chemicals for the same disease in an article, they will not be treated as a combination therapy unless the relation of the two chemicals is “AND”. For example, in comparison study among different medications, the relation of different medications is “OR” rather than “AND”, and thus will not be annotated as a combination therapy.

### Annotation tools

**Fig. 1:**
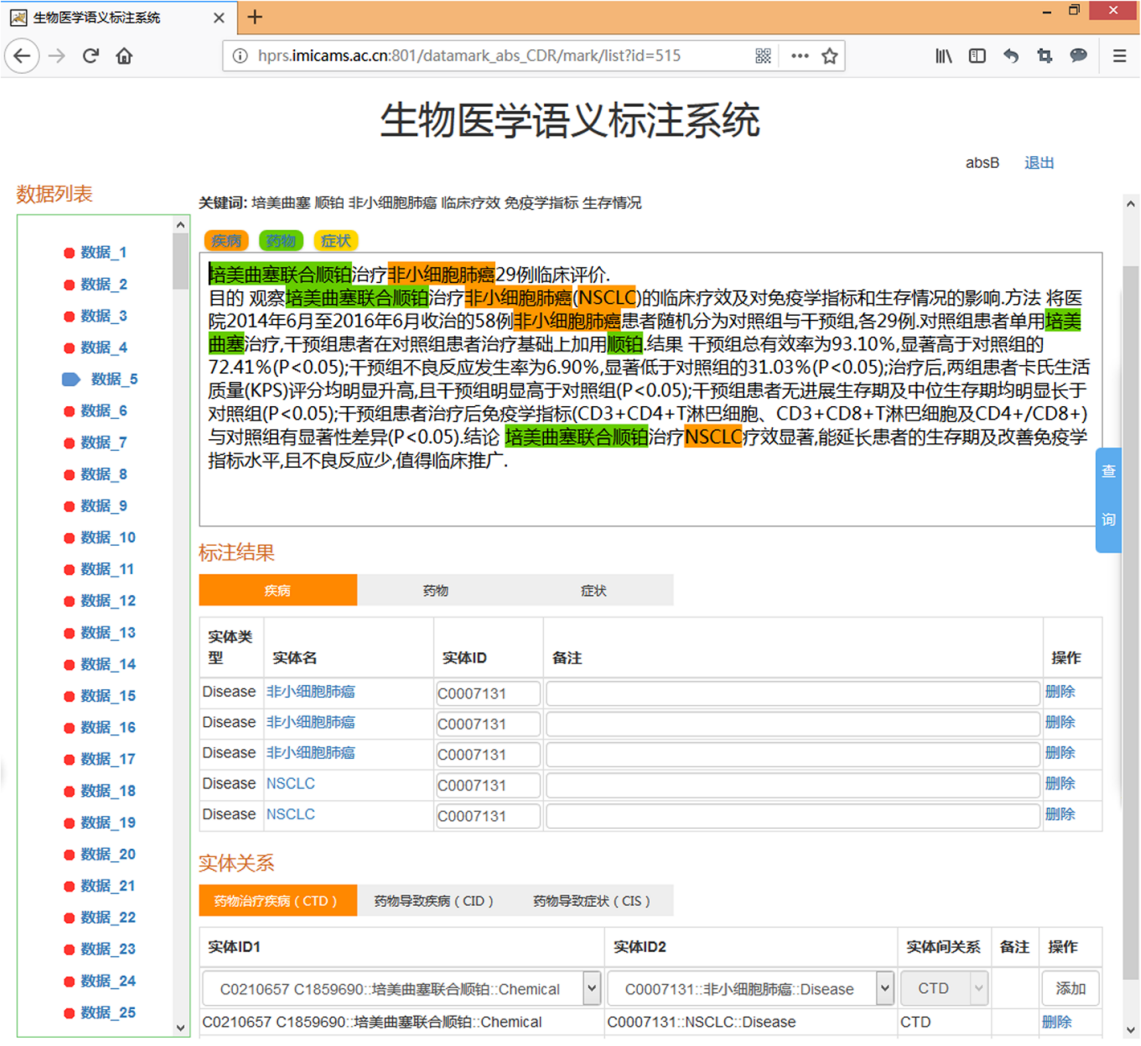
Annotation example shown in the annotation tool CBSAS

Manual annotation of disease and chemical entities was performed by using of the annotation system Chinese Biomedical Semantic Annotation System (CBSAS) which was developed according to the previous efforts in corpus annotation tool PubTator [20, 21]. Figure 1 shows an example in our annotation tool CBSAS. In CBSAS, the relationship annotation is followed by the entity annotation, and different entity types and relationships are separated by different tabs. Annotators can make notes in the remarks column during the annotation process.

Different with the BioCreative CDR annotation task, the annotators were asked to annotate the relations based on their own entity annotations rather than the gold-standard entity annotations, which was designed to improve the annotation efficiency.

### Annotation data formats

All annotation data is available in the PubTator format which consists of a straightforward tab-delimited text file. And two versions of annotations are provided: a version with relations with separate chemicals and a version with combination therapies. The annotators are required to provide only the version with combination therapies, and the annotation tool will automatically transfer it to the one with separate chemicals.

**Fig. 2:**
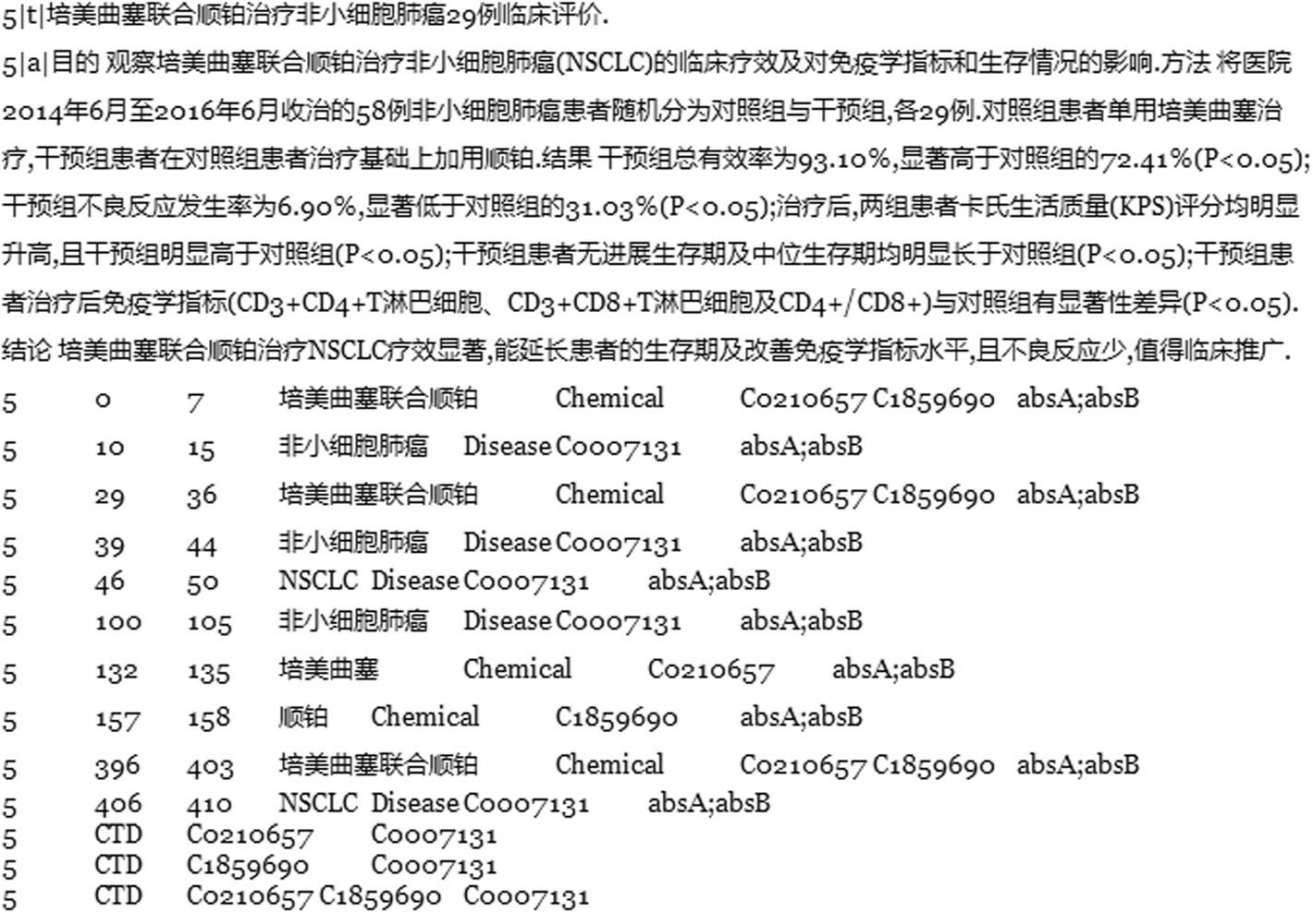
An example of annotation results of the combination therapy version

Figure 2 shows an example of the second version, which outputs the annotation result of the article with the title “Clinical Evaluation on Pemetrexed Combined with Cisplatin in Treating 29 Patients with Non-Small Cell Lung Cancer”. In the last line of the result, the relation is recorded as “5 CTD C0210657 C1859690 C0007131” rather than “5 CTD C0210657 C0007131” and “5 CTD C1859690 C0007131”. The system provides the comparison analysis different annotators which is recorded in the last cell of the annotation result lines.

### Inter-annotator agreement (IAA) analysis

To assess the consistency of the entity and entity relationship annotation, the metrics used are equivalent to others more commonly used in IE evaluations. We measured pairwise agreement of duplicate annotations using the F score where the independent annotations served as the benchmark set of the other one.

If an annotation has exactly the same article ID, start and end point and concept identifier with the benchmark set, then it will be counted as TP (True Positive). If an annotation exists in the annotations of team member while not in the benchmark set, then it will be counted as FP (False Positive). If an annotation exists in the benchmark set but does not match any in the annotations of team members, it will be counted as FN (False Negative). And P (Precision), R (Recall) and F score (F) are calculated as (1), (2) and (3) respectively.

1 $$ p=\frac{TP}{TP+ FP} $$

2 $$ R=\frac{TP}{TP+ FN} $$

3 $$ F=\frac{2\times TP}{2\times TP+ FP+ FN} $$

For the CTD task, if a CTD relation mention has exactly the same article ID, chemical ID, disease ID and relation type with the benchmark set, then it will be counted as TP. For a combination therapy, the system will omit the order of the chemical IDs. For example, “C0210657 C1859690” is as the same as “C1859690 C0210657”. Following the work of Roberts [22], a relaxed IAA will be evaluated in the future study. Also a relaxed matching F score is also used, in which the cases with same start/end point but different concept identifier are counted as TP. For relation mentions, a relaxed F score is computed based on a unified entity mention set.

## Results

### Corpus overview

The resulting corpus consists of 339 Chinese biomedical articles with 2367 annotated chemicals, 2113 diseases, 237 symptoms, 164 chemical-induce-disease relations (CID), 163 chemical-induce-symptom relations (CIS), and 805 chemical-treat-disease relations (CTD). For entity mentions, chemical mentions and disease mentions are much more than symptoms. For relation mentions, there are more CTD mentions than CID and CIS ones. It seems that the corpus is more available for chemical, disease and CTD recognition.

Since the topics of the dataset are predefined rather than randomly given, division of the dataset according to the topic distribution will be a reasonable choice. To help further training of the relation extraction models, the corpus is manually partitioned to a training set and a test set with a proportion of 4:1 to make the topic distributions of the two sets similar to each other. As shown in Table 2, the resulting two data sets have similar distributions of chemical mentions, chemical IDs, disease mentions, disease IDs, symptom mentions and CTD relations, which makes the corpus more useful for training models. The corpus contains more chemical than disease mentions, and contains more CTD relations than CID and CIS. And each article has at least one CTD relations, which indicates that the corpus is more applicable for the chemical-treat-disease relation recognition task.

**Table 2:** The overall corpus statistics

	Training	Test	Total
Articles	271	68	339
Chemical Mention	1870	497	2367
Chemical ID	455	135	526
Disease Mention	1711	402	2113
Disease ID	309	95	354
Symptom Mention	184	53	237
Symptom ID	72	32	84
CID	135	29	164
CIS	120	43	163
CTD	636	166	802

**Fig. 3:**
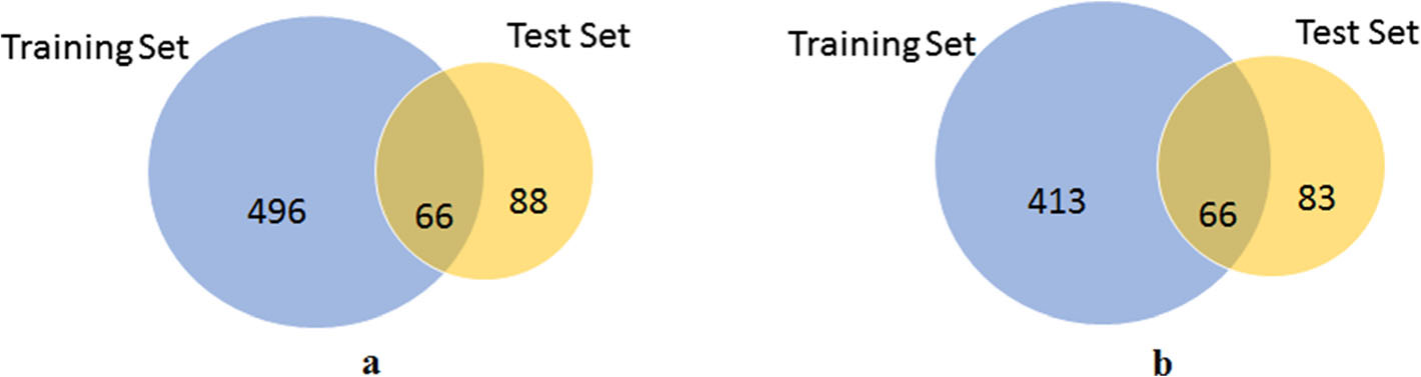
Distribution of chemical mentions and disease mentions in the corpus. **a** Chemical mentions distribution. **b** Disease mentions distribution. The blue circle is the number of unique concepts from the training set, the yellow circle is the number of unique concepts from the test set, and the light brown circle is the overlap of concepts from both of the two sets

**Fig. 4:**
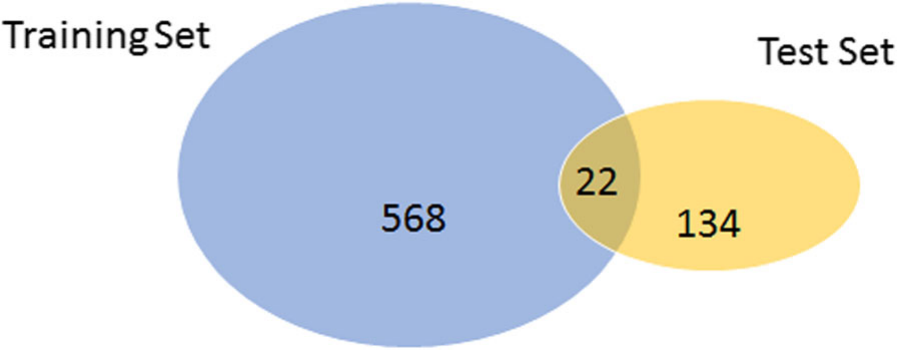
Distribution of CTD mentions in the corpus. The blue circle is the number of unique relations from the training set, the yellow circle is the number of unique relations from the test set, and the light brown circle is the overlap of relations from both of the two sets

By contrast with the overall statistics, we can identify several entity mention overlaps between the training data set and the test data set (see Fig. 3). Distributions of CTD mentions in the corpus are shown in Fig. 4. There are chemical and disease overlapping between training and test sets, which has a risk of over-fitting when training NER models. However, there are comparatively less interactions between the relation mentions which is similar to the relation mentions distribution of BioCreative CDR. Since our aim is to discover relations rather than entities, the risk of over-fitting is low.

### Inter-annotator agreement for mention annotation

Existing corpora often focus on the annotation of single entities and do not provide inter-annotator agreement scores. In our work, both of the entities and relation inter-annotator agreement scores are presented.

The results of inter-annotator agreement with the measure of F score and relaxed F score are presented in Table 3. The chemical mention, disease mention get higher inter-annotator agreement F scores, and the relations CID, CIS and CTD get relatively lower agreement scores. It is calculated that chemicals are easier to annotate (88.3% agreement) than diseases (79.1%) and symptoms (62.7%). This is probably due to the fact that most of the chemicals correspond to commercial drugs that are formed by a single token, while many diseases are expressed by multiword terms, giving rise to higher variation. Sometimes, it is rather difficult to differ a symptom from a disease in CMeSH, and hence, difficulty for their annotation.

**Table 3:** Inter-annotator agreement F scores of the corpus

Object	F	Relaxed F
Chemical	0.883	0.944
Disease	0.791	0.859
Symptom	0.627	0.765
CID	0.382	0.628
CIS	0.456	0.783
CTD	0.479	0.788

The relaxed F scores which omit the differences of concept identifiers are higher than the F scores, which indicate that a large proportion of disagreements are for the identifier discrepancies.

The relation IAAs indicate that the relation annotation work is more subjective than the entity annotation work. And that the pipeline workflow which annotates entities and relationships at the same time can easily cause cascading error especially for the relation annotation which may enlarge the disagreements produced during the period of entity annotation.

### Disagreement analysis

Discrepancies between the two independent annotators have been checked. For entity annotations, disagreements are concluded to two types: 1) Inconsistent boundaries including omitted mentions, wrong mentions and different boundaries. 2) Inconsistent concept IDs including error ID or different choices of IDs. For chemical mentions, 47.45% disagreements are boundary ones and 52.55% ID ones. Of the ID disagreements, the concept ID “-1”takes a proportion of 16.83% which indicate that the unknown chemical entries in the CMesh dictionary will influence the annotation quality. And it is noticed that the combination annotations which have two or more chemical IDs in an entry is more likely to get the boundary disagreements than the single ones (e.g. “硫酸沙丁胺醇溶液+布地奈德混悬液” (Salbutamol Sulfate Solution Combined with Budesonide Inhalation Solution), annotators have different ideas with whether “混悬液” (Inhalation Solution) should be included in the mention). For disease mentions, 57.41% disagreements are boundary ones and 42.59% ID ones. The concept ID “-1” takes only 5.17% in the disease concept ID disagreements which indicate that the CMesh dictionary coverage of disease is much better that that of chemicals. For relation annotations, a large proportion of discrepancies are for the inconsistent entity annotations, in other words, the efficiency of the pipeline workflow is at the expense of accuracy.

## Discussion

### A comparison with other related works

Comparing to other related works on the annotation and corpus building of the CDRs (Table 4), there are three main characteristics in this study. Firstly, our corpus is the only CDR corpus of biomedical articles in Chinese, which can be further applied in the text mining tasks targeted at biomedical texts in Chinese. Secondly, our topics are focused on specific chronic diseases, and combination therapies information is curated and expressed in the CDRs for the first time, which will facilitate researchers to extract combination therapy related knowledge. Thirdly, we tried a pipeline annotation workflow in which annotators annotate the entities and relations at the same time. The workflow improves the annotation efficiency and may provide more hints for training a joint model for NER and relation extraction, however, results of the disagreement analysis shows that the pipeline workflow approach causes much more discrepancies among different annotators and may result in lower inter-annotator agreements scores for relations.

**Table 4:** A comparison of works on the corpus building of CDRs

Corpus or author name	Language	Dictionary	Sources	Scale	Text boundary	Annotation results
Roberts [11]	en	UMLS	clinical text	150	sentence	Condition, intervention, drug, locus and their interaction relations
i2b2/VA [12]	en	–	clinical text	871	sentence	Relation types that hold between medical problems, tests, and treatments
EU-ADR [13]	en	MeSH/UMLS++ [23]	abstracts	300	sentence	Drugs, disorders, targets and their inter-relationships
Rosario [14]	en	MeSH	abstracts	3495 sentences	sentence	Relationships between treatment and disease
IxaMed-GS [24]	spa	SNOMED CT	Clinical text	75 docs/5410 sentences	document	Relationships between entities indicating adverse drug reaction events
BioCreative CDR [16]	en	MESH [25]	abstracts	1500	document	Relationships between chemicals and diseases (CID)
RCorp	cn	CMESH [19]	abstracts	339	document	Relationships between chemicals and diseases (CTD)

### Limitations and future studies

In this study, the topics of the articles were limited and thus limited the applications of the corpus. And the combination therapy related relations, especially for CID and CIS relations, are not sufficient enough more training models. Our next step is to enlarge the annotation scope and size. To improve the agreement rates, we will change to two-phase approach for the entity annotation and relation annotation as work in [16, 24]. And an evaluation of relation extraction will be held in the future. We hope that the corpus serve as an important resource for developing relation extraction tools which automatically mine relations from biomedical abstracts in Chinese.

## Conclusions

In this study, we demonstrated a new annotation work for chemical disease semantic extraction in Chinese. The corpus is chronic disease specific and targeted at combination therapy related mining from biomedical abstracts in Chinese. The result analysis of the corpus proves its quality for the chemical-treat-disease relation identification task. Our annotated corpus would be a useful resource for the modelling of relation extraction tools. In the future, we will further enlarge the size of the corpus, and use it to evaluate related semantic relation tools which will be applied in information providing platforms to enhance the visualization of biomedical texts and help knowledge graph construction.

## Data Availability

Since the datasets in this study will be applied in an evaluation for further study. To make the evaluation reasonable and fair for all the participants, the datasets are not publicly available now. And all the datasets will be opened for te public after the evaluation.

## Abbreviations

CBSAS: Chinese Biomedical Semantic Annotation System
CCKS: China Conference on Knowledge graph and Semantic computing
CDR: Chemical-disease relations
CHV: Consumer health vocabularies
CID: Chemical-induce-disease relation
CIS: Chemical-induce-symptom relation
CMeSH: Chinese Medical Subject Headings
CTD: Chemical-treat-disease relation
EU-ADR: a group to exploit different European electronic healthcare records (EHR) databases for drug safety signal detection
I2B2: The Informatics for Integrating Biology and the Bedside
IAA: Inter-annotator agreement
IE: Information extraction
NER: Named entity recognition
NLP: Natural language processing
RCorp: Relation Corpus
SNOMED-CT: Systematized Nomenclature of Medicine-Clinical Terms
UMLS: Unified medical language system
